# Chemical Diversity and Defence Metabolism: How Plants Cope with Pathogens and Ozone Pollution

**DOI:** 10.3390/ijms10083371

**Published:** 2009-07-30

**Authors:** Marcello Iriti, Franco Faoro

**Affiliations:** Università degli Studi di Milano, Dipartimento di Produzione Vegetale, Sezione di Patologia Vegetale, Via Celoria 2, 20133 Milano, Italy

**Keywords:** stress physiology, secondary metabolism, phytochemicals, phytoalexins, phytoanticipins, tropospheric ozone pollution, volatile organic compounds

## Abstract

Chemical defences represent a main trait of the plant innate immune system. Besides regulating the relationship between plants and their ecosystems, phytochemicals are involved both in resistance against pathogens and in tolerance towards abiotic stresses, such as atmospheric pollution. Plant defence metabolites arise from the main secondary metabolic routes, the phenylpropanoid, the isoprenoid and the alkaloid pathways. In plants, antibiotic compounds can be both preformed (phytoanticipins) and inducible (phytoalexins), the former including saponins, cyanogenic glycosides and glucosinolates. Chronic exposure to tropospheric ozone (O_3_) stimulates the carbon fluxes from the primary to the secondary metabolic pathways to a great extent, inducing a shift of the available resources in favour of the synthesis of secondary products. In some cases, the plant defence responses against pathogens and environmental pollutants may overlap, leading to the unspecific synthesis of similar molecules, such as phenylpropanoids. Exposure to ozone can also modify the pattern of biogenic volatile organic compounds (BVOC), emitted from plant in response to herbivore feeding, thus altering the tritrophic interaction among plant, phytophagy and their natural enemies. Finally, the synthesis of ethylene and polyamines can be regulated by ozone at level of S-adenosylmethionine (SAM), the biosynthetic precursor of both classes of hormones, which can, therefore, mutually inhibit their own biosynthesis with consequence on plant phenotype.

## 1. Introduction

In their ecosystem, plants have to cope with a plethora of potentially unfavourable conditions. Stress factors affecting plant fitness not only derive from natural sources, such as adverse temperature fluctuations (heating, chilling, freezing), high irradiation (photoinhibition, photooxidation), osmotic imbalance (salinity, drought), hypoxia/anoxia (flooding), mineral (macro- and micronutrient) deficiency, wounding, phytophagy and pathogen attack, but also from anthropogenic activities. The latter include xenobiotics employed in agriculture (pesticides), environmental (air, soil and water) pollutants and increased UV radiation.

**Figure 1.**
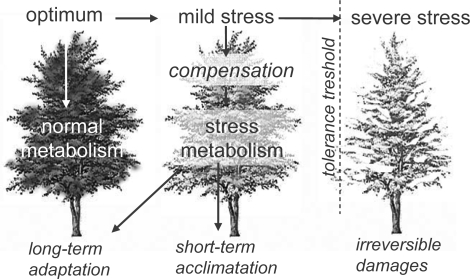
Stressor dose-stress effect relationships in plants.

In any case, regardless of natural or anthropogenic stress factor, plants have to cope with their stressors. From a pathophysiological point of view, a plant may avoid or adapt to a particular stress with a dose-dependent mechanism (Figure 1). Under a certain threshold, a mild stress may be compensated by the plant, whereas, at higher levels, the detrimental effects of a severe stress may cause irreversible damages, according to the stressor dose-stress effect relationship [1]. Furthermore, the stress tolerance threshold depends not only on the type of stressor and exposure time, but also on the plant stress-coping capacity. In this view, the shift between normal and stress metabolism represents a fundamental trait in plant acclimation (short-term) and adaptation (long-term) strategies, although is almost impossible to define exactly the threshold between them [2].

In this survey, we deal with the secondary metabolites involved in plant resistance against pathogens and tolerance to ozone, a widespread atmospheric pollutant, emphasising on metabolic fluxes between primary and secondary metabolism induced by both biotic and abiotic stresses.

## 2. Plant Secondary Metabolism

In living organisms, secondary metabolites are usually considered not essential for growth and development, unlike the products of primary metabolism. This does not mean they are not important, as they relate plants with the components of their ecosystem, i.e., the physic environment (biotope) and the living community (biocenosis), thus resulting indispensable for the survival of the species. Moreover, it must be pointed out that the widely accepted division of metabolism in “primary” and “secondary” is somewhat unsatisfactory. In fact, some major chemical groups still classified as secondary metabolites, such as lipids, are required by all living organisms, as are some metabolites arising from a secondary pathway (i.e., giberellin A_1_ from the isoprenoid pathway) that can have a determinant endogenous regulator role [3] Additionally, stress metabolism could be regarded as a particular expression of secondary metabolism, when stressful conditions, of both biotic and abiotic nature, change the dynamic equilibrium of the ecosystem. As an instance, phytoalexins are compounds synthesized *ex novo* or whose synthesis increase after pathogen challenging, raising their tissue concentration [4].

In plants, chemical diversity has determined their evolutionary success. Because of their sessile habitus, plants are unable to avoid worsening environmental conditions, and they cannot escape the plethora of previously mentioned biotic stresses. Consequently, unlike animals, plants have evolved an enormous number of secondary metabolites to overcome any danger, though most of them are species-specific. This implies that the actual number of secondary metabolites within a given species is much more limited. The functional role of these phytochemicals range from the ecology to defence, improving protection against both biotic and abiotic stresses, besides being involved in ecological roles as attractants or repellents, for pollinators and phytophagy respectively, colours and scents of reproductive organs (flowers and fruits).

Generally, precursors of secondary metabolic pathways are products of the primary metabolism. Therefore, a severe or long-lasting stress factor could induce an excessive shift between primary and secondary metabolism and, consequently, a diversion of essential available resources from growth to defence. To a large extent, secondary metabolites derive from three biosynthetic routes, namely the phenylpropanoid, isoprenoid and alkaloid pathways. Phytochemicals arising from these pathways include not only compounds with a broad-spectrum antibiotic activity, but also powerful antioxidants able to counteract oxidative stress [5–7].

### 2.1. Phenylpropanoid Pathway

**Scheme 1.**
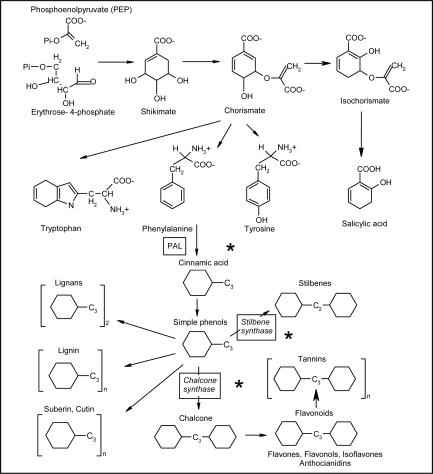
Aromatic amino acid biosynthesis from shikimate and phenyl-alanine derivatives of the phenylpropanoid pathway. Depending on the species and the conditions (i.e., during pathogen attack) salicylic acid can also be synthesized from chorismate.

Phenylpropanoids are a class of phenylalanine derivatives with a basic C_6_−C_3_ (phenyl-propane) skeleton (Scheme 1). In turn, the essential amino acid phenylalanine arises from the shikimate pathway, as well as the other aromatic amino acids tyrosine and tryptophan. Precursors of this pathway are phosphoenolpyruvate, from glycolysis, and erythrose 4-phosphate from pentose phosphate pathway, leading to two important intermediates, shikimic and chorismic acid. In further steps, after a branch point, phenylalanine and tyrosine are synthesized from prephenic and arogenic acid, whereas tryptophan from anthranilic acid [8].

**Scheme 2.**
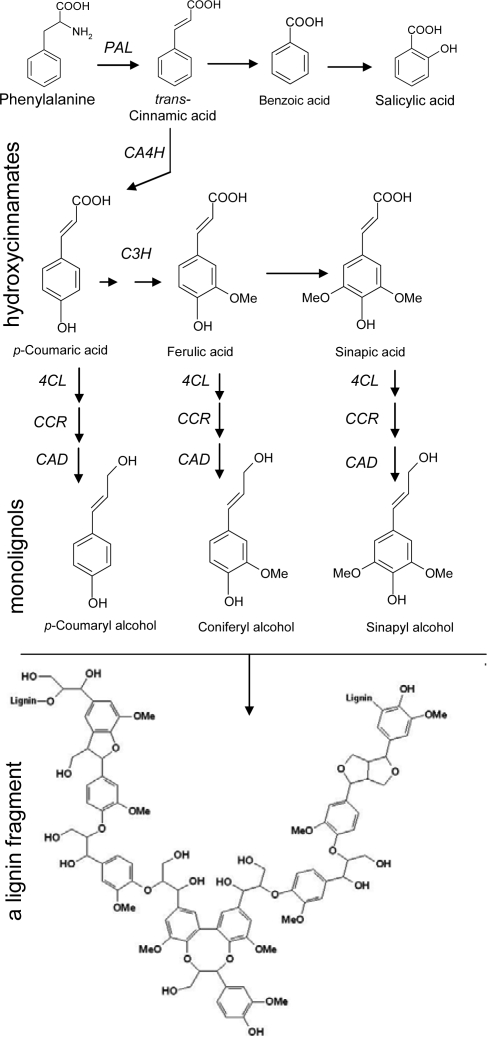
Main steps of the phenylpropanoid pathway leading to benzoic acids, hydroxycinnamates (coumaric, ferulic and sinapic acids) and lignin, (CA4H, cinnamate-4-hydroxylase; C3H, coumarate-3-hydroxylase; CAD, cinnamyl alcohol dehydro-genase CCR, cinnamoyl: CoA reductase; CL, 4-Coumarate: CoA ligase; PAL, phenylalanine ammonia-lyase.

The removal of an amino group from phenylalanine (deamination) via the step catalyzed by the enzyme phenylalanine ammonia-lyase (PAL), leads to cinnamic acid and, in turn the precursor of hydroxycinnamates after a series of hydroxylation of the benzene ring (Scheme 2). These compounds, including coumaric, ferulic and sinapic acids are reduced to the corresponding alcohols via aldehyde intermediates, namely coumaryl, coniferyl and sinapyl alcohol, collectively termed monolignols.

Dimerization or polymerization of monolignols leads to lignans and lignin, respectively (Scheme 2). Lignification is a complex reaction in which peroxidases catalyze the polymerization of lignin units, consuming H_2_O_2_. Apposition of lignin in plant cell wall is a process occurring during the development of particular tissues, as well as in plant defence response, in order to strengthen the cell wall and protect the plasmalemma. Benzoic and hydroxybenzoic acids (C_6_−C_1_), such as salicylic acid (SA), a molecule importantly involved in systemic acquired resistance (SAR), represent another group of cinnamic acid derivatives, formed by cleavage of a C_2_ fragment from the phenylpropane structure (Scheme 2) [7,9]. However, it must be pointed out that, depending on the species and the conditions (i.e., during pathogen attack), salicylic acid can also be synthesized directly from chorismate-isochorismate by isochorismate synthase [10].

The metabolites mentioned till now can be collectively named simple phenols, to differentiate them from polyphenolic compounds. The latter have an additional benzene ring, arising from the cyclization of three malonic acid (C_2_) residues via stilbene synthase (STS) or chalcone synthase (CHS), to give stilbenes (C_6_−C_2_−C_6_) or flavonoids (C_6_−C_3_−C_6_), respectively (Scheme 1). Finally proanthocyanidins (PA), or condensed tannins, include oligo- and polymeric flavonoid derivatives (from flavanols catechins), with polymerisation degree ranging from 2 to 17 and more (Scheme 1). The numerous hydroxyl groups available in these molecules promote the formation of complexes with macromolecules, such as proteins and polysaccharides or metal ions [7–9].

### 2.2. Isoprenoid Pathway

Isoprenoids, also named terpenoids, represent the chemically and functionally most diversified class of low molecular mass lipids in plants, both primary and secondary metabolites [11]. They include electron carriers (quinones), membrane constituents (sterols), vitamins (A, D, E and K), plant hormones (side chain of cytokinins, abscisic acid, gibberellins and brassinosteroids), photosynthetic pigments (chlorophyll, phytol and carotenoids) and essential oils [12].

Acetyl coenzyme A (CoA) represents the precursor for the isprenoid biosynthesis (Scheme 3). Firstly, two molecules of acetyl CoA react to give acetoacetyl CoA and, then, with a further acetyl CoA to produce β-hydroxy-β-methylglutaryl(HMG)-CoA. In plants, the same enzyme, HMG-CoA synthase, catalyses both reactions. The conversion of HMG-CoA into mevalonate, via HMG-CoA reductase, is the rate limiting enzyme of this pathway [13]. Mevalonate kinase and mevalonate phosphate kinase phosphorylate, respectively, mevalonate and, then, mevalonate 5-phosphate, yielding mevalonate 5-diphosphate. Afterwards, mevalonate diphosphate decarboxylation, via mevalonate diphosphate decarboxylase, produces isopentenyl diphosphate (IPP), the five-carbon building block for the formation of isoprenoid chains. The enzyme IPP:dimethylallyl-PP isomerase converts IPP into dimethylallyl diphosphate (DMAPP), the acceptor for successive transfers of isopentenyl residues [13,14].

Hemiterpenes (C_5_), such as isoprene, originate from dimethylallyl-PP, upon the release of diphosphate. Differently, dimethylallyl-PP can condense with IPP, to form geranyl-PP via geranyl-PP synthase. In the same way, further chain elongation is attained by head to tail condensation of geranyl-PP to IPP, to produce farnesyl-PP via farnesyl-PP synthase. Analogously, geranylgeranyl-PP synthase catalyses the head to tail condensation of farnesyl-PP to IPP, thus yielding geranylgeranyl-PP [15]. Geranyl-PP is the precursor for the formation of monoterpenes (C_10_) or essential oils, including highly volatile open chain and cyclic compounds, such as menthol, limonene, geraniol, linalool and pinene. They are active in plant-microbe, plant-pronubi, plant-phytophagous and plant-plant interactions, due to their attractiveness or repulsiveness [16]. Farnesyl-PP is the precursor for the synthesis of open chain and cyclic sesquiterpenes (C_15_), the largest group of isoprenoids, including essential oils and antibiotic compounds (phytoalexins) [17].

Diterpenes (C_20_) derive from geranylgeranyl-PP, consisting of phytoalexins, plant hormones, the phytol side chain of chlorophylls, tocopherols and phylloquinone [18].

Additionally, triterpenes (C_30_) are synthesized from two molecules of farnesyl-PP (C_15_), by a reductive head to head condensation. The triterpene squalene is the precursor for sterols, important membrane constituents, via squalene synthase [19]. Analogously, head to head condensation of two molecule of geranylgeranyl-PP (C_20_) leads to tetratepenes (C_40_), such as the carotenoids (carotene, lycopene) and xanthophylls (lutein, zeaxanthin, violaxanthin). Besides, isoprenoids are involved in protein prenylation, that is the synthesis of variously lengthened isoprenoid chains anchoring proteins in membranes, such as G proteins, ubiquinone, plastoquinone and cytochrome-a. Finally, natural rubber is a polyterpen, composed of over 1,000 isoprene units and deriving from polymerisation of geranylgeranyl-PP units [20].

### 2.3. Alkaloid Pathways

**Figure 2.**
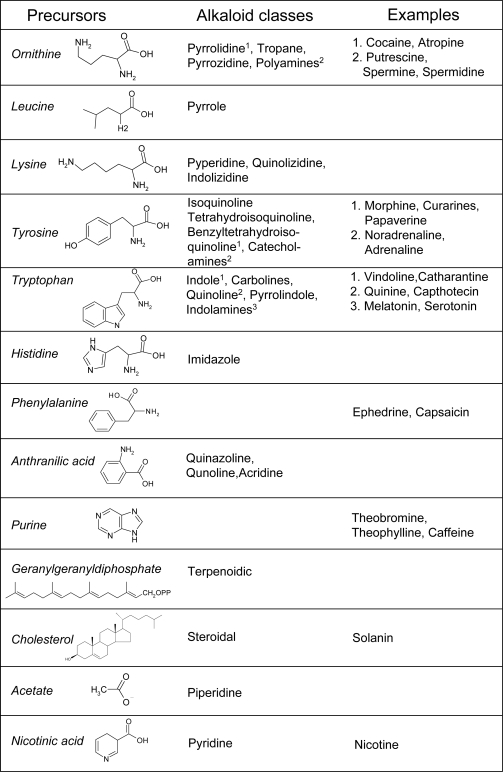
Main classes of alkaloid precursors and derivatives (the example number refers to the corresponding alkaloid class).

A common pathway for alkaloid biosynthesis does not exist. A majority of alkaloids are amino acid derivatives grouped on the basis of their precursor and chemical structure. Therefore, the main groups include alkaloids arising from ornithine, leucine, lysine, tyrosine, tryptophan, histidine and phenylalanine, in addition to alkaloids arising from nicotinic (pyridine alkaloids) and anthranilic acid, acetate, isoprenoids and purine (Figure 2). In turn, these classes include some minor divisions. Accordingly, pyrrole alkaloids arise from leucine; pyrrolidine, tropane and pyrrolizidine alkaloids from ornithine; piperidine, quinolizidine and indolizidine alkaloids derive from lysine; catecholamines, isoquinoline, tetrahydroisoquinoline and benzyltetrahydroisoquinoline alkaloids originate from tyrosine; indolamines, indole, carboline, quinoline, pyrrolindole and ergot alkaloids come from tryptophan and imidazole alkaloids from histidine. Anthranilic acid is the precursor of quinazoline, quinoline and acridine alkaloids, whereas isoprenoid alkaloids include mono- (geraniol), di- (geranylgeranyl-PP) and triterpene (cholesterol) derivatives (Figure 2) [5,21,22].

Alkaloids constitute an enormous number of phytochemicals of toxicological, pharmacological, nutritional and cosmetic interest, and of ecological importance for plants. For instance, tropane alkaloids include cocaine and atropine, nicotine is a pyridine alkaloid, noradrenaline (or norepinephrine), adrenaline (epinephrine), papaverine, curarines and morphine arise from tyrosine. Melatonin and serotonin are indolamines, vindoline, catharantine (and their derivatives vincristine and vinblastine) are indole alkaloids, quinine and capthotecin are quinoline alkaloids and lysergic acid diethylamide (LSD) is an ergot alkaloid, all these arising from tryptophan. Histamine is an imidazole alkaloid, ephedrine and capsaicin derive from phenylalanine, solanin is a steroidal glycoalkaloid from cholesterol. Finally, purine alkaloids include theophylline, theobromine and caffeine, found in tea, cacao and coffee, respectively [5,21,22].

## 3. Secondary Metabolites Involved in Plant Resistance against Pathogens

Higher plants are regularly exposed to microorganisms, at the level of both epigeous and hypogeous organs. However, only a few of them causes diseases, because of an efficient plant innate immune system. Therefore, as for animals, compatibility of micro-organisms with a susceptible plant represents an exception in nature, while incompatibility with a resistant host is the rule. The latter condition depends on the plant defence machinery, a complex array of both physical and chemical preformed and inducible defences [23]. Plant antimicrobial compounds are broadly classified into two categories: phytoanticipins and phytoalexins. The former are low molecular weight compounds present in plant before micro-organism challenge, or produced after infection from pre-existing precursors, while the latter are both synthesized and accumulated in plant after exposure to micro-organisms or abiotic agents [24].

### 3.1. Phytoanticipins

Plant preformed antimicrobial compounds (phytoanticipins) include three important classes: saponins, cyanogenic glycosides and glucosinolates [25].

**Scheme 4.**
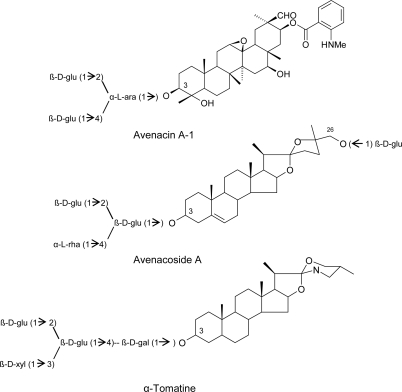
Molecular structures of the major oat and tomato saponins (adapted from Osbourn, [32]).

Saponins are glycosylated compounds widely distributed among plant families and divided into three major groups, depending on the structure of the aglycone, which may be a triterpenoid, a steroid or a steroidal glycoalkaloid. The genus *Avena* is particularly rich in these metabolites, with oat containing two different families of saponins, the triterpenoid avenacins, in roots, and the steroid avenacosides in leaves, both groups mainly concentrated in the epidermal layers (Scheme 4). In turn, avenacosides are closely related to the steroidal glycolakaloid α-tomatine, occurring in tomato (Scheme 4). Saponins destabilize plasmalemma integrity by forming complexes with membrane sterols and causing pore formation. Plants avoid these toxic effects by compartmentalizing them in the cell vacuole or in other organelles, whose membranes are protect because of a low or different sterol composition [26–28]. Resistance of oat to the root-infecting fungus *Gaeumannomyces graminis* pv tritici, the causal agent of the take all disease in wheat and barley, has been attributed to the presence of avenacins in oat roots [29–32]. Conversely, the oat-infecting *G. graminis* pv avenae is able to tolerate and detoxify avenacins by secreting extracellular glycosidases (avenacinases) which remove the terminal sugar from their carbohydrate chain, thus altering the amphiphatic properties of these saponins [31,33,34]. In tomato, the content of α-tomatine has been correlated with the resistance to the tracheomycosis (vascular diseases) caused by *Fusarium oxysporum* pv lycopersici and *Verticillium albo-atrum*, as well as with the foliar pathogen *Cladosporium fulvum* [35–37].

**Scheme 5.**
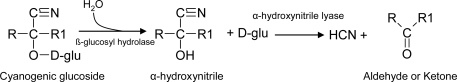
Generation of hydrocyanic acid from a cyanogenic (HCN) glucoside precursor. The α-hydroxynitrile intermediate is then converted by α-hydroxynitrile lyase to HCN and aldehyde or ketone.

Cyanogenic glucosides are phytoanticipins known to be present in more than 2,500 plant species. They are regarded as having an important role in plant defense against herbivores due to bitter taste and release of toxic hydrogen cyanide (HCN) upon tissue disruption (reviewed in [38]). In fact cyanogenic glycosides are precursors of HCN, a respiratory poison released after the cleavage of the carbohydrate moiety by a β-glucosyl hydrolase (or simply a β-glucosidase; Scheme 5).

The corresponding α-hydroxynitrile intermediate is then converted to HCN by an α-hydroxynitrile lyase, associated with defence against pathogens and phytophagy (Scheme 5) [39]. Healthy cyanogenic plants do not contain detectable amounts of HCN, because, in their tissues, cyanogenic glycosides are normally compartmentalized, i.e., spatially separated from the enzymes that catalyse HCN production. Important cyanogenic glycosides include dhurrin and amygdalin, from sorghum and almond respectively [40–42]. Pathogens of cyanogenic plants produce the cyanide-inducible enzyme cyanide hydratase, which detoxify HCN by converting it to formamide [43].

**Scheme 6.**
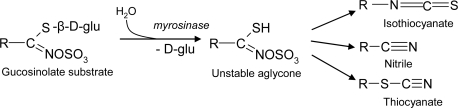
Hydrolysis of glucosinolates and degradation of the unstable aglycone to isothiocyanates, nitriles and athiocyanates.

Glucosinolates are sulphur-containing glucosides widespread in members of the family Cruciferae, including the genus *Brassica*, of relevant agronomic interest, and the weed Arabidopsis, a model plant in molecular genetic and biology. They represent a large chemical family including over 120 different compounds with known defensive properties against herbivores [44,45]. Glucosinolates can be subdivided into three major classes, depending on their side chain, which may be derived from aliphatic (methionine), indolyl (tryptophan) or aralkyl (phenylalanine) α-amino acids. Within individual plants, their distribution is tissue specific, as in oilseed rape, where aliphatic glucosinolates abound in leaves, whereas indolyl and phenylethyl glucosinolates predominate in roots and stems. Like cyanogenic glycosides, these compounds are also activated in response to tissue damage by the action of myrosinase (thioglucoside glucohydrolase or simply thioglucosidase), an enzyme that, in healthy plants, is separated from its glucosinolate substrates by subcellular compartmentalization (Scheme 6). The unstable aglycone thus generated may then form various products, including nitriles, thiocyanates and volatile isothiocyanates, being the latter the major and the most fungitoxic glucosinolate breakdown products (Scheme 6) [39,46,47]. Therefore, high levels of glucosinolates have also been associated with plant (*Brassica*) resistance to pathogens, such as the blackleg fungus *Leptosphaeria maculans* and the oomycete *Peronospora parasitica*, causal agents of stem canker and downy mildew, respectively [48,49]. Inhibition of ascospores of *Mycosphaerella brassicae* has been early reported, in both cabbage and cauliflower leaves [50] Interestingly, indolyl glucosinolates may be used as precursors for the biosynthesis of auxin, a indole/tryptophan derivative, by fungi that cause hyperplasic and hypertrophic tissue growth, such as the root gall fungus *Plasmodiophora brassicae* [37].

### 3.2. Phytoalexins

The recognition of elicitors by the plant’s own perception repertoire represents a crucial stimulus to initiate phytoalexin synthesis. Elicitors are (exogenous) signal molecules of microbial origin, or generated endogenously from plant cell wall fragments produced at the host-pathogen interface. In any case, plant necessitates of receptors in order to recognize the pathogen and mount its own defence reaction [23]. Interestingly, phytoalexins accumulate both in resistant and susceptible hosts at the same concentrations, though with a different kinetic, thus pointing out that their efficacy strictly depends on the timing of their synthesis at the infection site [51].

Activation of plant defence response generally induces an intense, though transient, diversion of normal (primary) metabolism into synthesis of phytoalexins, secondary metabolites *de novo* synthesized from essential substrates such as phenylalanine, acetyl-CoA, malonyl-CoA, mevalonic acid or other amino acids. Phytoalexins have been identified in the majority of plant families, and members of a plant family normally produce similar types of phytoalexins, though phenylpropanoids include several classes of well-studied phytoalexins, for instance isoflavonoids from the Leguminoseae family [52,53].

The *Phaseolus vulgaris-Colletotrichum lindemuthianum* pathosystem has provided a good model for studying the role of phytoalexins in plant resistance against pathogen attack. *C. lindemuthianum*, the causal agent of bean anthracnose, is a hemibiotrophic fungus whose colonization is restricted, in resistant hosts, because of the production of isoflavonoid phytoalexins, including phaseollin, phaseollin isoflavan and kievitone [28,54]. Similar examples include medicarpin and pisatin, two isoflavonoid phytoalexins from alfalfa (*Medicago sativa*) and pea (*Pisum sativum*), respectively [55,56]. However, broad bean (*Vicia faba*) provides a notable exception. Like most other legumes, it produces isoflavonoid phytoalexins, but the principal induced antimicrobial compounds are furanoacetylenic wyerone derivatives [28,52].

In the Vitaceae family, the phytoalexins which have been well characterized constitute a rather restricted group of molecules belonging to the stilbene family, synthesized as a general response to fungal attack. These compounds possess the skeleton based on the trans-resveratol (3,5,4′-trihydroxy-stilbene) structure, including piceids, pterostilbenes and viniferins, that are, respectively glucosides, dimethylated derivatives and oligomers of resveratrol [57]. In grapevine (*Vitis vinifera*), the activities of CHS and STS are differentially regulated, according to the plant developmental stage (see section 2.1). During the initial phase of berry ripening (véraison), resveratol accumulation in cells of berry skin declines, while anthocyanin synthesis increases, due to the competition between the two branches of the same pathway. As a consequence, since véraison, anthocyanin accumulation confers colour to berry skin, whereas the lowering levels of the powerful phytoalexin resveratrol make bunches more susceptible to *Botrytis cinerea* infections (grey mould) [59]. Interestingly, open field treatments with plant activators (compounds able to activate the plant immune system) can reverse, to a certain degree, the inverse relationship between resveratrol and anthocyanin content, reducing the CHS and STS competition for the substrate and avoiding the metabolic switch from one to the other pathway. Thus, higher levels of resveratrol protect grape from grey mould after véraison, without hampering the colouring phase, which is an important qualitative trait [59].

**Scheme 7.**
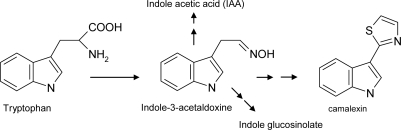
Schematic biosynthetic pathway of camalexin via indole-3-acetoaldoxime. Other important indolic compounds arise from the same route.

In the model plant Arabidopsis, camalexin (3-thiazol-2′-yl-indole) (Scheme 7) represents the main phytoalexin, involved in inducible defence mechanisms against a variety of pathogens, such as the bacterium *Pseudomonas syringae* and the fungus *Alternaria brassicicola*. Camalexin is a N-and S- containing indole phytoalexin synthesized from tryptophan via indole-3-acetaldoxime, a branch point metabolite that also leads to the biosynthesis of the previously described glucosinolates, besides the plant hormone indole acetic acid (IAA) and melatonin (Scheme 7) [60].

Finally, it is important to underline that phytoalexin (and phytoanticipin) synthesis is only a component of the plant co-ordinated defence strategy, contributing to restrict the pathogen spreading together with structural barriers, oxidative burst, hypersensitive response (HR) and pathogenesis related (PR) proteins [23].

## 4. Tropospheric Ozone and Plant Health

**Figure 3.**
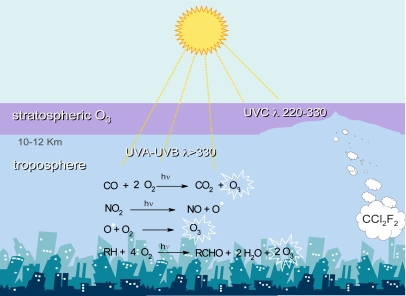
Beneficial and detrimental pools of ozone in the stratosphere and troposphere, respectively. In the stratosphere this pool is depleted by fluorochlorocarbons (CCl_2_C_2_) while in the troposphere it is raised by photochemical smog.

In the atmosphere, two different pools of O_3_ exists, the beneficial and the detrimental one (Figure 3). In the stratosphere (the higher atmosphere, ranging approximately from 15 to 40 km in altitude), the ozone layer absorbs the harmful UV-B and UV-C radiations, thus screening the living organisms [61,62]. Conversely, in the troposphere (the lower part of the atmosphere, approximately from the Earth surface to 10-12 km in altitude), ozone is regarded as a pollutant [63]. Tropospheric ozone is a secondary pollutant produced through reactions between primary pollutants, emitted directly into the air (mainly nitric oxides, sulphur oxides, carbon oxides and hydrocarbons), catalyzed by sunlight (Figure 3) [64]. Consequently, ozone is produced on bright sunny days over areas with intense primary pollution, due mainly to vehicle exhausts, fossil fuel burning and industrial processes, in the so-called photochemical cycle [65,66].

Ozone enters the leaf tissues through the stomata and plants are exposed to either acute and chronic ozone doses, according to the gas concentration and exposure time. Because of its strong oxidizing potential (+ 2.07 eV), O_3_ is a powerful oxidizing agent capable of reacting with virtually any biomacromolecule, including lipids, proteins, nucleic acids and carbohydrates, although it is neither a radical species nor a ROS [67,68]. In cell membrane, polyunsaturated fatty acids represent the primary target for ozone, stimulating lipid peroxidation and impairing membrane fluidity [69]. Modification of proteins can occur, both in their structure and activity, and DNA damage can also be produced, as shown in animals by the increased activity of poly(ADP-ribose) synthetase (PARP), a chromatin-bound enzyme promoting damaged DNA repair [70,71]. Though the increase of PARP activity has not been so far investigated in plants after ozone exposure, it is likely to occur as a general defence response, similar to that observed in Arabidopsis after pathogen attack [72].

Symptoms of injury are generally unifacial and visible in the adaxial (upper) leaf surface, depending on leaf morphology. The most common symptoms due to chronic injury include chlorosis (yellowing due to the chlorophyll breakdown, often distributed in spots over the leaf) and bronzing (red-brown pigmentation caused by phenylpropanoid accumulation), while lesions attributable to acute exposures are much more diversified. In broad-leaved plants, they include bleaching (small unpigmented necrotic spots), flecking (small brown necrotic areas fading to grey or white), stippling (small punctuate spots, white, black or red in colour) and tipburn (dying tips, firstly reddish, later turning brown) specially in conifers [73,74].

At a physiological level, ozone exposure impairs stomatal function and reproductive development, decreases photosynthetic activity and CO_2_ photoassimilation, and induces senescence, resulting in dysfunction of transpiration and water use efficiency, reduction of dry matter production and yield losses [75–79].

### 4.1. Ozone and Phenylpropanoids

In plants, phenylpropanoid metabolism is induced as a general response to stress. Therefore, enhancement of key enzyme activities and accumulation of secondary metabolites are events occurring early after challenging, in order to improve the resistance against pathogen attack and/or tolerance to adverse environmental conditions and pollutants. PAL is an extremely sensitive indicator of stress conditions, and it is commonly considered as a biochemical marker indicating the activation of plant defences, including the synthesis of both structural and protective compounds. In particular, ozone exposure elevates the level of flux through the phenylpropanoid pathway, thereby supplying carbon skeletons for secondary metabolites [80].

The enhancement of phenylpropanoid biosynthesis by ozone is well documented. A very early report, dated more than 30 years ago, documented the accumulation of isoflavonoids in soybeans following ozone exposure [81]. Ever since, ozone-stimulated induction of transcripts for defence-related genes, the same induced by pathogens, as well as the increased activity of key enzymes of the phenylpropanoid pathway have been reported in several plant systems. In Arabidopsis (*Arabidopsis thaliana*), PAL mRNA is rapidly and transiently induced within 3 h of ozone treatment (300 ppb daily for 6 h), reaching a 3-fold higher levels than control plants [82]. A similar trend has been reported in parsley (*Petroselinum crispum*) plants, in which ozone treatment (200 nL L^−1^ for 10 h) induced an early 3-fold and 1.2-fold increase of PAL and CHS activity, respectively, followed by a 2-fold increase of total leaf furanocoumarins and flavone glycosides [83]. The content of psoralen, bergapten and other furanocoumarins in celery (*Apium graveolens*) dropped 24 h after ozone (0.2 ppm for 2 h) fumigation, but levels of these chemicals, in treated leaves, increased rapidly at 120 h [84]. Further studies have shown the overlap between patterns of genes induced by ozone exposure and pathogen infection, probably due to the role of ROS as effector molecules involved in transduction pathways activated either by pathogens and ozone. The enhancement of SA content, in plant tissues, due to ozone treatment is well documented [85]. Intriguingly, in tobacco (*Nicotiana tabacum*) plants, a pulse ozone treatment (120–170 nmol mol^−1^ for 5 h) enhanced the emission of methyl salicylate, a volatile SA derivative, to a greater extent in sensitive cv. Bel W3 compared with tolerant cv. Bel B [86]. In Arabidopsis, SA accumulation, necessary for the expression of hypersensitive response to pathogens (HR) and SAR, is also required for the accumulation of some ozone-induced mRNAs, particularly PAL and pathogenesis related protein 1 (PR1) transcripts. Interestingly, ozone-induced PAL mRNA accumulation was unaffected in plants expressing salicylate hydroxylase, demonstrating that the ozone induced accumulation of PAL transcripts does not depend on SA accumulation [88]. These findings demonstrate that ozone activates at least two distinct signaling pathways, including a salicylic acid dependent pathway previously shown to be associated with the activation of pathogen defense reactions, and a second one SA-independent, as a protective response to ozone. [88]. HR is a type of programmed cell death (PCD) triggered at the attempted pathogen penetration site, frequently at the onset of systemic immunity (SAR) [83,84], whereas PR proteins are enzymes induced in plants by pathogen infection, as well as by abiotic and environmental stresses, included ozone [89–93]. In sensitive bean (*Phaseolus vulgaris*) cv. Pinto, ozone exposure (120 nL L^−1^ for 4 h) causes a stimulation of phenylpropanoid route and flavonoid branch, as shown by the increased mRNA accumulation of PAL, CHS and chalcone isomerase (CHI), the latter involved in isoflavonoid biosynthesis [93]. In grapevine (*Vitis vinifera*), STS, the first enzyme of the stilbene branch involved in the synthesis of resveratrol and other stilbenic compounds, is considered the most sensitive ozone-induced biomarker [94]. In grapevine callus, either PAL and STS activity increase, after ozone fumigation (0.3 μmol mol^−1^ for 2 h), unlike CHS [90]. This could be due to the STS and CHS competition for the same substrate (Scheme 1), as it happens during the véraison, previously described. Furthermore, a general drop in the amount of some assayed phenylpropanoids (coumaric acid, ferulic acid, gallic acid and catechin) has been reported, unlike caffeic acid, whose level raised only in one cell line [95]. In 20 soybean (*Glycine max*) cultivars, ozone tolerance has been associated with the presence of kaempferol glycosides, a powerful antioxidant flavonol, as well as tolerance to manganese (Mn) toxicity in one soybean line [96]. In European silver birch (*Betula pendula*) chronically exposed to ozone, a 16.2% increase in totals phenylpropanoids and a corresponding 9.9% increase of 10 compounds, among simple phenols and flavonoids, such as chlorogenic acid and catechin, respectively, has been reported [92]. Interestingly, the combined action of CO_2_ and ozone greatly enhances the synthesis of total and polymeric PA, in *Betula* sp. leaf tissues, suggesting an additive effect of these environmental pollutants on phenylpropanoid biosynthesis [98].

Shikimate dehydrogenase (SKDH), a key enzyme of shikimate pathway, PAL and cinnamyl alcohol dehydrogenase (CAD), a key enzyme of lignin biosynthesis which forms monolignols (Scheme 2), have been investigated in poplar (*Populus tremula* x *alba*) leaves. Under ozone exposure (60–120 nL L^−1^, during the 14 h light period, for 1 month), either CAD activity and transcript levels have been rapidly and strongly stimulated, increasing up to 15-fold and 23-fold the control values, respectively. In contrast, SKDH and PAL activity raised only in old and middle-aged leaves, but not in the youngest ones. Interestingly, the increased activity of these enzymes has been associated with a higher lignin content in ozone-exposed leaves and, additionally, the newly synthesized lignin structurally differed from the control lignin. Particularly, stress lignin appeared more condensed, i.e., enriched in carbon-carbon interunit linkages, in *p*-hydroxyphenyl (H) units and in terminal units with free phenolic groups (Scheme 2) [99]. The same enzymes have been studied in two genotypes of ozone-treated (150 nL L^−1^ for 3 h) tomato (*Lycopersicon esculentum*) plants [100]. However, while SKDH and PAL activity augmented significantly only in one line, CAD activity diminished in both the genotypes, in contrast with results reported by other authors [99,101]. An explanation could reside in the different response of herbaceous and woody plants and in the different acute or chronic ozone dose employed in tomato and poplar, respectively.

To conclude, the importance of phenylpropanoids in plant tolerance against ozone injury is related to their different properties. These compounds include an array of molecules with a plethora of biological activities, besides precursors of structural biopolymer, such as lignin. Particularly, their protective role is mainly inherent to their antioxidant power, i.e., the ability to trap free radicals (ROS), functioning as electron donors. Nonetheless, increased synthesis of lignin, as well as structural modifications of lignin itself, represent another important defence mechanism, in order to protect plasmalemma from the ROS injury, thus preventing membrane damages due to lipid peroxidation [7].

### 4.2. Ozone and Isoprenoids

Strictly speaking, not all the isoprenoids are secondary metabolites, as some primary metabolites, such as sterols, arise from the isoprenoid pathway as well. The effects of the pollutant on either the sterol concentration and composition have been early reported in several plants [102–104]. Sterols are important component of cell membranes, involved in their stabilization. Generally, exposure to high ozone concentrations (>12 μL L^−1^) results in a decrease of free sterols (FS) and an increase in bound sterols (BS). Conversely, at low ozone concentration (<12 μL L^−1^) an accumulation of FS occurs, resulting in a decrease of the FS:BS ratio. In this case the enhancement of FS synthesis is followed by a shift towards sterols with a more bulky C-17 side chain, i.e., sitosterol and stigmasterol vs. campesterol. As FS have a much greater capacity to stabilize membranes than BS, the FS:BS ratio has a more determinant effect on membrane permeability than the composition of FS fraction itself. Accordingly, decrease in FS:BS ratio with high ozone concentration results in cell injury and visible damages, whereas modification of FS composition, occurring at lower ozone levels, results neither in permanent membrane injury nor in visible damages [105–107]. In tobacco leaves, ozone fumigation resulted in increased lipid concentrations, but decreased levels of FS and triglycerides [103], whereas a higher amount of total phytosterols has been reported in fumigated plants by other authors [108].

In ozone treated (0.3 μL L^−1^ for 8 h) Scots pine (*Pinus sylvestris*), it has been reported a transient increase of a transcript corresponding to the cytosolic/endoplasmic HMG-CoA synthase, a key enzyme of isoprenoid biosynthesis [109]. The C_5_ precursor of isoprenoids, IPP, arises either from mevalonate pathway, in the cytosol, as previously described, or from plastidial precursors [110]. In ozone-treated pine seedlings (250 nL L^−1^, 12 h day^−1^ for 4 days), the biosynthesis of plastidial IPP has been inhibited, differently from the mevalonate synthesis in the cytosol, due to the resource allocation between the two IPP synthesis pathways [111].

**Figure 4.**
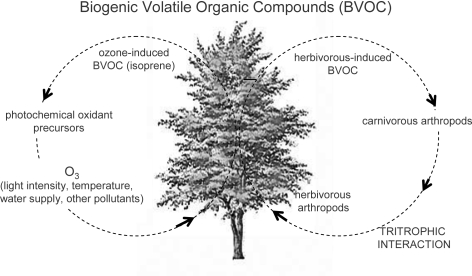
Relationships between ozone and herbivorous-induced BVOCs and their influence on photochemical ozone precursors and on tritrophic interactions, respectively.

Biogenic volatile organic compounds (BVOC) comprise mainly isoprenoids (particularly hemi-, mono- and sesquiterpenes) emitted from plants during cell growth and in response to several kinds of stresses, thus having various ecophysiological functions and mediating plant-arthropod interactions (Figure 4) [112]. BVOC can act as attractants for pollinators or repellents for noxious insects, besides being involved in tritrophic signalling, i.e., the relationship between plant, herbivorous and carnivorous arthropods. In particular, phytophagus feeding can induce BVOC emission from plants, which can function as foraging cues for the recruitment of the natural enemies of herbivores [113], and for the orientation, landing and feeding of other phytophagy [114]. Besides wounding and trithrophic interactions, several environmental factors can affect the BVOC emission from plants, such as light intensity, temperature, water supply and pollutants (Figure 4) [115]. The relationships between ozone and BVOC is somewhat complex, both exerting a mutual influence [116]. On the one hand, the release of either BVOC and volatile organic compounds (VOC) from anthropogenic origin into the atmosphere can constitute a significant input of photochemical oxidant precursors, thus contributing to the regional-scale air pollution; on the other hand BVOC emission can be triggered by the exposure to high ozone concentration [86,117]. Additionally, chronic ozone exposure can modify the composition of the plant BVOC emissions [118–121], thus not only affecting the tritrophic interactions, but also directly weakening plant defence responses against arthropods [122]. Isoprenoids can be synthesized and emitted in order to tolerate the ozone injury. Isoprene, a hemiterpene (Scheme 3), has been reported to reduce ozone damages in leaves, because of its antioxidant activity. This gas protects the photosynthetic apparatus, quenches ozone byproducts and radical species responsible for lipid peroxidation of cell membranes and cell death [123–125]. Besides, monoterpenes may exert an isoprene-like antioxidant activity, too [126,127].

### 4.3. Ozone and Alkaloids

The effect of ozone on alkaloid biosynthetic pathways has not been extensively investigated, although a generalized influence of the pollutant on the nitrogen metabolism has been ascertained [128–130]. Generally, ozone exposure reduces the amount of total alkaloids in tobacco plants [128,129], and lower levels of nicotine, a pyridine alkaloid, in ozone exposed plants have been related to increased survival, growth and development of hornworm (*Manduca sexta*) larvae [130]. Several studies reported increased preferences or enhanced survival and fitness of different insect species on ozonated plants, thus pointing out again the detrimental effect of the pollutant on the plant chemical defences [132–136].

**Scheme 8.**
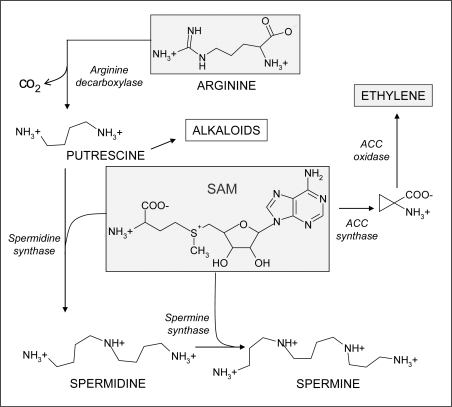
Involvement of S-adenosylmethionine (SAM) in polyamine and ethylene biosynthesis. The metabolic shift to ethylene or polyamine biosynthesis can enhance ozone susceptibility or tolerance, respectively.

The role of polyamines, important alkaloid precursors (Scheme 8), has been correlated with ozone tolerance. Polyamines are polycationic nitrogenous compounds of low molecular weight ubiquitous in all living organisms. In plants, they function as growth regulators involved in an array of physiological processes, being involved in embryogenesis, cell division, morphogenesis, development, flowering and senescence [137]. In addition, they serve as an integral component of plant response to both biotic and abiotic stresses [138,139]. The most important polyamines are the diamine putrescine (Put), the triamine spermidine (Spd) and the tetramine spermine (Spm), arising directly from the free, non proteinogenic amino acid ornithine, by ornithine decarboxylase (ODC), or indirectly from arginine by arginine decarboxylase (ADC). Further steps include Put conversion into Spd, via spermidine synthase (SPDS), and the similar synthesis of Spm from Spd via spermine synthase (SPMS). Either these enzymes employed the aminopropyl moiety provided by S-adenosylmethionine (SAM) [140]. In plants, ADC activity is enhanced by ozone exposure, whereas the ODC one remaines unchanged [141,142]. Free and conjugated polyamines improve ozone tolerance with two different mechanisms: by inhibiting the ethylene biosynthesis and by direct ROS scavenging, respectively [143,144]. Ethylene and polyamines share the same biosynthetic precursor (SAM) and, thereby, they mutually inhibit their own biosynthesis. In particular, aminocyclopropane carboxylic acid (ACC), a precursor of ethylene, arises from SAM via ACC synthase, a rate-limiting step in the ethylene production. Therefore, this metabolic shift to ethylene or polyamine biosynthesis can enhance ozone susceptibility or tolerance, respectively, due to the correlation between the stress ethylene production and visible ozone injury [142]. Additionally, apoplastic polyamines can form conjugates with hydroxycinnamates and phenolic acid derivatives, effective in ROS detoxification [145]. As reported above, polyamine are precursors of alkaloids, thus, apart they role in ozone tolerance, higher amounts of these compounds, as a consequence of ozone exposure, could induce an increase of pyrrolidine, tropane and pyrrolizidin alkaloids, deriving from ornithine via Put.

## 5. Conclusions

Chemical diversity determined the evolutionary success of plant organisms, favouring their adaptation to a changing environment. Plants cope with a plethora of stressful abiotic and biotic conditions by modifying their secondary metabolic pathways [146]. In this view, chemical diversity improved, during the evolution, the fitness of plant organisms, thus ensuring their evolutionary radiation [147]. Phytochemicals, with their broad spectrum activities, are primarily involved in plant tolerance towards worsening climatic conditions and environmental pollutants, as well as in resistance against pests and pathogens. The metabolic processes activated in these defence responses may be tightly separated or overlapping, according to the stress factor and the plant cultivar, as a result of negative (*trade off*) or positive (*cross resistance*) cross talk, respectively, i.e., the communication between molecular signals and transduction pathways involved in different plant defence responses. A cross tolerance may also occur, in plant, from a cross induction, that is the ability to alert the defence mechanisms against an abiotic stress in consequence of priming with a biotic elicitor and vice versa.

Therefore, the influence of atmospheric pollution in plant-pathogen interactions is quite complex [148,149]. On the one hand, ozone may induce the same sequel of events involved in plant innate immunity [23], i.e., oxidative burst and hypersensitive response at the onset of systemic acquired resistance (SAR) [88], on the other hand plants weakened by ozone stress my be particularly susceptible to infections [149]. Interestingly, wheat rust (*Puccinia recondita* f. sp. *tritici*) was strongly inhibited by ozone, but was unaffected by elevated CO_2_, both in presence or absence of ozone stress [150]. Vice versa, a protective effect of rust (*Uromyces fabae*) infection in broad bean (*Vicia faba*) was reported against ozone, sulphur dioxide either alone or combined [151].

**Figure 5.**
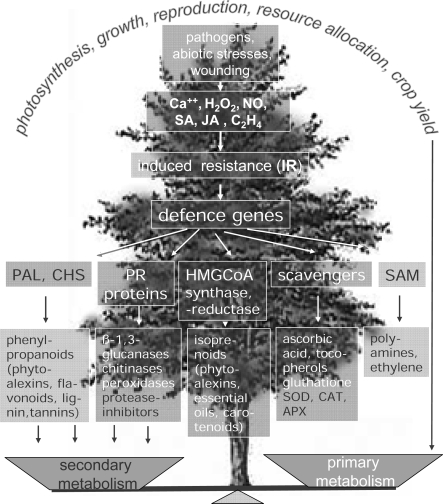
Influence of different stresses on plant metabolism. The activation of induced resistance leads to the accumulation of numerous defence compounds that may unbalance the equilibrium between primary and secondary metabolism, thus resulting in fitness costs for the plant. On the other hand, the synthesis of secondary metabolites due to a stress may protect plant from other different stresses.

Finally, to the other side of the coin, an excessive production of secondary metabolites may be detrimental for the plant’s fitness (Figure 5), in terms of allocation costs and autotoxicity [152,153], though it may be favourable, at least in food plants, which could provide foodstuffs and beverages enriched of bioactive phytochemicals [154].

## Figures and Tables

**Sckeme 3.**
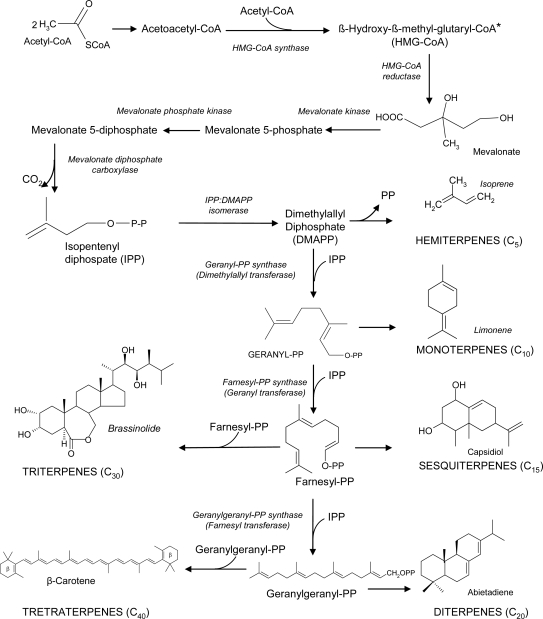
Isoprenoid pathway from acetyl-CoA. The enzyme with an asterisk is reported to be influenced by ozone.
